# Functional analysis of the eTM-miR171-*SCL6* module regulating somatic embryogenesis in *Lilium pumilum* DC. Fisch

**DOI:** 10.1093/hr/uhac045

**Published:** 2022-02-19

**Authors:** Rui Yan, Shengli Song, Hongyu Li, Hongmei Sun

**Affiliations:** Key Laboratory of Protected Horticulture of Education Ministry, College of Horticulture, Shenyang Agricultural University, National and Local Joint Engineering Research Center of Northern Horticultural Facilities Design and Application Technology, Shenyang 110866, China; School of Agriculture, Ningxia University, Yinchuan, Ningxia 750021, China; Key Laboratory of Protected Horticulture of Education Ministry, College of Horticulture, Shenyang Agricultural University, National and Local Joint Engineering Research Center of Northern Horticultural Facilities Design and Application Technology, Shenyang 110866, China; College of Life Science and Bioengineering, Shenyang University, Shenyang 110866, China; Key Laboratory of Protected Horticulture of Education Ministry, College of Horticulture, Shenyang Agricultural University, National and Local Joint Engineering Research Center of Northern Horticultural Facilities Design and Application Technology, Shenyang 110866, China

## Abstract

Somatic embryogenesis (SE) is of great significance in *Lilium* bulb production, germplasm preservation, and genetic improvement. miRNAs are important regulators of plant growth and development at the transcriptional level. Previous research by our group has shown that lpu-miR171 and its target gene *SCARECROW-LIKE 6* (*SCL6*) play an important regulatory role in lily SE, and we predicted and identified that endogenous target mimics (eTMs) can regulate lpu-miR171. However, the associated mechanism and internal regulatory network are not yet clear. In the present study, lpu-miR171 was used as an entry point to explore the regulatory network between its upstream eTMs and its downstream target gene *LpSCL6*, as well as to identify the mechanism of this regulatory network in *Lilium* SE. Tobacco transient transformation confirmed that miRNA171 significantly inhibited the expression of *LpSCL6*. On this basis, the *Lilium* stable genetic transformation system was used to demonstrate that silencing lpu-miR171a and lpu-miR171b and overexpressing *LpSCL6-II* and *LpSCL6-I* promoted starch accumulation in calli and the expression of key cell cycle genes, thus providing energy to meet preconditions for SE and accelerate the formation and development of *Lilium* somatic embryos. LpSCL6-II and LpSCL6-I are nuclear proteins with self-activation activity in yeast cells. In addition, we confirmed in *Lilium* that lpu-eTM171 is the eTM of lpu-miR171 that binds lpu-miR171 to prevent cleavage of the target gene *LpSCL6*, thereby promoting SE. Therefore, the present study established a new mechanism whereby the eTM-miR171-*SCL6* module regulates SE in *Lilium pumilum* and provides new insights clarifying the mechanism of SE.

## Introduction

*Lilium* species are among the most important ornamental plants and economic crops worldwide. This genus includes some of the most important cut flower species, potted plants, bedding plants, and garden crops, and the bulbs of this genus may be edible or medicinal. The improvement of genetic traits and the cultivation of high-quality bulbs are key, difficult problems in the field of international *Lilium* production and biotechnology. Somatic embryogenesis (SE) in plants, which presents the advantages of universal occurrence, genetic stability, a low mutation rate, and a high reproduction coefficient, is vital for bulb propagation, germplasm preservation, genetic trait improvement, mutant creation, and breeding by genetic engineering in *Lilium* [1]. However, due to the high specificity and poor synchronization of somatic embryo induction in different genotypes, the requirements for mass breeding and genetic engineering research in *Lilium* cannot be met, mainly because the mechanism of SE is not yet clear.

MicroRNAs (miRNAs) are a class of endogenous, small non-coding RNAs ~19–24 nucleotides in length. To date, 38 589 miRNAs have been published in the miRBase database (Release 22.1) [2]. Additionally, a large number of miRNA target genes have been identified by bioinformatics, omics, and immunoprecipitation, as well as other methods. As regulatory factors of endogenous gene expression, miRNAs have been widely recognized to play an important role in SE in a variety of plants, such as rice [3], citrus [4, 5], larch [6–8], cotton [9], maize [10], and longan [11, 12]. Specifically, miR156-*SPL* modules play a vital role during the initial phases of SE induction [5]. Overexpression of csi-miR156a or suppression of one of the two target genes, *CsSPL3* and *CsSPL14*, can enhance the SE competence of citrus callus. miR159/*LaMYB33* is involved in the maintenance of embryogenic ornon-embryogenic potential and somatic embryo maturation in larch [7]. miR166/*ATHB15* regulates early somatic embryogenesis and miR167a/*ARF8* regulates globular embryo formation and subsequent conversion to cotyledonary embryos in longan [11]. Similar stages of SE in different plants or different stages of SE in the same plants are regulated by different miRNAs or multiple miRNAs simultaneously. The molecular mechanism of miRNA regulation of SE needs further analysis.

Our previous study found that miR171 family members are differentially expressed in torpedo embryos and cotyledonary embryos compared with globular embryos in *Lilium pumilum*. Subsequently, we cloned miRNA171 and its target gene and preliminarily confirmed that miRNA171 plays an important role in torpedo embryo development in *Lilium pumilum* [13]. However, the molecular mechanism by which miR171 regulates *Lilium* SE has not been elucidated. The members of the miR171 family are 21 nucleotides long and are highly conserved in angiosperms [14]. miR171 regulates various plant growth and development processes by targeting members of the *GRAS* domain transcription factor family, which are essential for the maintenance of shoot apical meristems and axillary meristems [15]. A series of studies have shown that miR171 participates in the regulation of SE by modulating the *SCL6* target gene. miRNA171 is one of the key miRNAs in the process of SE, as observed in larch [6], radish [16], citrus [4], and longan [12], and it has been shown to play an important regulatory role in the different stages of SE. In *Larix kaempferi* SE, 11 conserved miRNA families were detected by quantitative reverse transcription–PCR (qRT–PCR), among which miR171a/b might have proembryogenic functions, while miR171c acts in the induction process of larch SE [6]. In larch, the cleavage site of miR171 and the *SCL6* target gene was identified through 5′ RACE (rapid amplification of cDNA ends) and through differential expression analysis, and it was shown that the posttranscriptional regulation of the *SCL6* target gene by miR171 is related to the maintenance of embryonic potential [8]. In *Citrus sinensis*, the expression patterns of miR171 and the *SCL6* target gene at different stages of somatic embryo development were analyzed, and the authors found that these patterns are closely related to the formation of somatic embryos [4]. In a study of *Raphanus sativus* L., miR171 accumulated in the early stage of SE, indicating that miR171 and the *SCL6* target gene play a key regulatory role in the early stage of SE [16]. The above studies show that miR171 and the *SCL6* target gene have an important regulatory function in SE; however, there are certain differences in their regulatory roles in the somatic embryos of different species. Their regulatory mechanism in SE has not been thoroughly analyzed, and there is little research on SE in lily. Current research directions have not included a deep analysis of the mechanism of miR171 in somatic embryos.

Since Franco-Zorrilla *et al*. first reported that the targeting activity of *Arabidopsis* miR399 is inhibited by endogenous target mimics (eTMs) [17], researchers have successively identified eTMs that regulate the activities of miR160, miR166, miRX27, and miR167 in plants such as *Arabidopsis* [18], rice [19], tomato [20], and longan [11]. The regulatory effect of eTMs on miRNAs has been verified in the processes of soybean lipid metabolism [21], tomato yellow leaf curl virus defense [22], and tobacco nicotine synthesis [23]. In addition, eTMs participate in a variety of plant development processes by regulating miRNAs. Regarding the regulation of SE, Lin *et al*. revealed the regulatory effects of miR160 and miR167 on auxin signaling in SE in longan by studying the differential expression levels of the miR160 and miR167 eTMs and the *ARF* target gene [11, 24]. However, the mechanism by which the eTM-miRNA module regulates SE has not been fully resolved.

In the early stage of our research, we used somatic embryos at different developmental stages as materials to construct the expression profiles of key miRNAs in SE. Although there are 13 members in the lily miR171 family, only miR171a and miR171b were differentially expressed during the somatic embryogenesis of *L. pumilum*, and the expression of the remaining mir171 members did not change significantly, with the expression level being extremely low [25]. We found that miR171a and miR171b are significantly differentially expressed during SE and that their expression peaks in embryogenic calli and during anaphase of SE [13]. RNA ligase-mediated (RLM) 5′ RACE has been used to verify the sites of action of lpu-miR171a and lpu-miR171b and the target genes, and a correlation analysis of the expression of lpu-miR171a and lpu-miR171b and the target genes in different stages of somatic embryo development has revealed that lpu-miR171a and lpu-miR171b have negative regulatory effects on *LpSCL6-II* and *LpSCL6-I.* To further clarify the mechanism by which lpu-miR171a and lpu-miR171b regulate SE, preliminary screening combined with bioinformatics methods was performed within the scope of the transcriptome to obtain standard eTMs. An expression correlation analysis of lpu-miR171a and lpu-miR171b and their corresponding eTMs revealed a negative correlation of the expression trends of lpu-miR171a and lpu-miR171b and their eTMs during SE, indicating that lpu-miR171 regulates target genes and is also regulated by the eTMs of upstream regulators [26]. Based on these previous studies, the present study explored the functions of lpu-miR171 and the *LpSCL6* target gene in the process of SE in *L. pumilum* through stable overexpression and the use of artificial miRNA silencing technology to elucidate the regulatory relationship of lpu-miR171 with the *LpSCL6* target gene. Overexpression and point mutation analyses confirmed the regulatory effect of eTM171 on lpu-miR171. Finally, the regulatory mode of eTM-miR171-*SCL6* in the process of *Lilium* SE was clarified, laying a foundation for the comprehensive elucidation of the mechanism of *Lilium* SE.

## Results

### Verification of the effects of lpu-miR171a on *LpSCL6-II* and lpu-miR171b on *LpSCL6-I*

**Figure 1:**
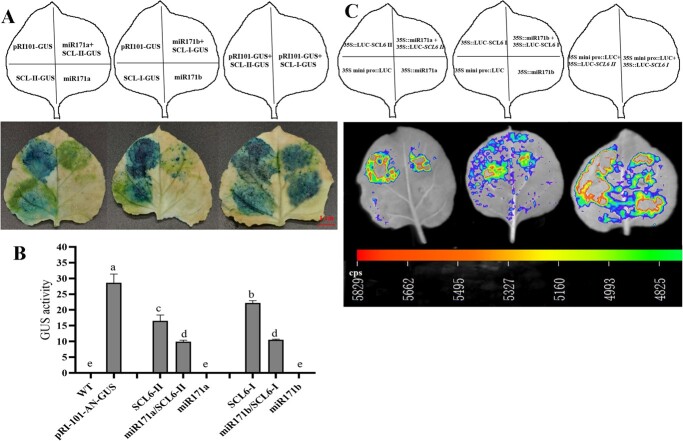
Interaction verification between lpu-miR171a and lpu-miR171b and the *LpSCL6-II* and *LpSCL6-I* target genes. **a** GUS histochemical staining. pRI101-GUS refers to the empty vectors. miR171a is a vector containing mature miR171a. miR171b is a vector containing mature miR171b. SCL-I-GUS is a vector containing *LpSCL6-I*. SCL-II-GUS is a vector containing *LpSCL6-II*. Scale bar = 1 cm. **b** GUS enzyme activity. **c** Verification results of lpu-miR171a and lpu-miR171b and the *LpSCL6-II* and *LpSCL6-I* target genes. 35S mini pro::LUC refers to the empty vectors. 35S::miR171a is a vector containing mature miR171a. 35S::SCL6 II is a vector containing *LpSCL6-II*. 35S::miR171a + 35S::SCL6 II refers to the observation result of the mixed injection of a vector containing mature miR171a and a vector containing *LpSCL6-II*. 35S::miR171b is a vector containing mature miR171b. 35S::SCL6 I is a vector containing *LpSCL6-I*. 35S::miR171b + 35S::SCL6 I refers to the observation result of the mixed injection of a vector containing mature miR171b and a vector containing *LpSCL6-I*. 35S mini pro::LUC + 35S::SCL6 I refers to the observation result of the mixed injection of empty vectors and a vector containing *LpSCL6-I*. 35S mini pro::LUC + 35S::SCL6 II refers to the observation result of the mixed injection of empty vectors and a vector containing *LpSCL6-II*.

The overexpression vectors of lpu-miR171a, lpu-miR171b, *LpSCL6-II*, and *LpSCL6-I* were transiently transfected into tobacco leaves, and pRI101-ON-GUS was used as a control. GUS histochemical staining was performed 48 h after transfection. Fig. 1a shows that tobacco leaves containing the *LpSCL6-II* and *LpSCL6-I* vectors and the pRI101-ON-GUS empty vector were stained dark blue. No blue color was observed in *Nicotiana benthamiana* leaves transformed with the lpu-miR171a and lpu-miR171b vectors without the GUS reporter gene or the tobacco leaves of the wild-type control group. Tobacco leaves cotransformed with lpu-miR171a and lpu-miR171b and the *LpSCL6-II* and *LpSCL6-I* target genes showed less intense staining. Measurement of GUS activity in the tobacco leaves demonstrated that GUS enzyme activity was higher in tobacco leaves containing the *LpSCL6-II* and *LpSCL6-I* vectors and the empty vectors. GUS enzyme activity was not detected in tobacco leaves containing the lpu-miR171a and lpu-miR171b vectors without the GUS reporter gene or the wild-type control group, while GUS enzyme activity was significantly reduced in tobacco leaves cotransformed with lpu-miR171a + *LpSCL6-II* and lpu-miR171b + *LpSCL6-I* (Fig. 1b). The above results indicated that lpu-miR171a and lpu-miR171b have an inhibitory effect on *LpSCL6-II* and *LpSCL6-I*, thereby preventing the expression of the GUS gene. The LUC luminescence signal results are shown in Fig. 1c. No LUC luminescence signal was observed from the vectors containing the lpu-miR171a and lpu-miR171b precursors or the empty pRI-mini35S-LUC vectors. 35S::LUC-SCL6 I and 35S::LUC-SCL6 II transfected alone produced a stronger LUC luminescence signal, while the LUC luminescence signal of cotransformed tobacco leaves was weaker. These results showed that lpu-miR171a and lpu-miR171b have an inhibitory effect on *LpSCL6-II* and *LpSCL6-I*, thereby reducing the LUC luminescence signal. Based on the above test results, we demonstrated that the lpu-miR171a and lpu-miR171b candidates have negative regulatory effects on *LpSCL6-II* and *LpSCL6-I*.

### LpSCL6-II and LpSCL6-I subcellular localization and transcriptional activation analysis

**Figure 2:**
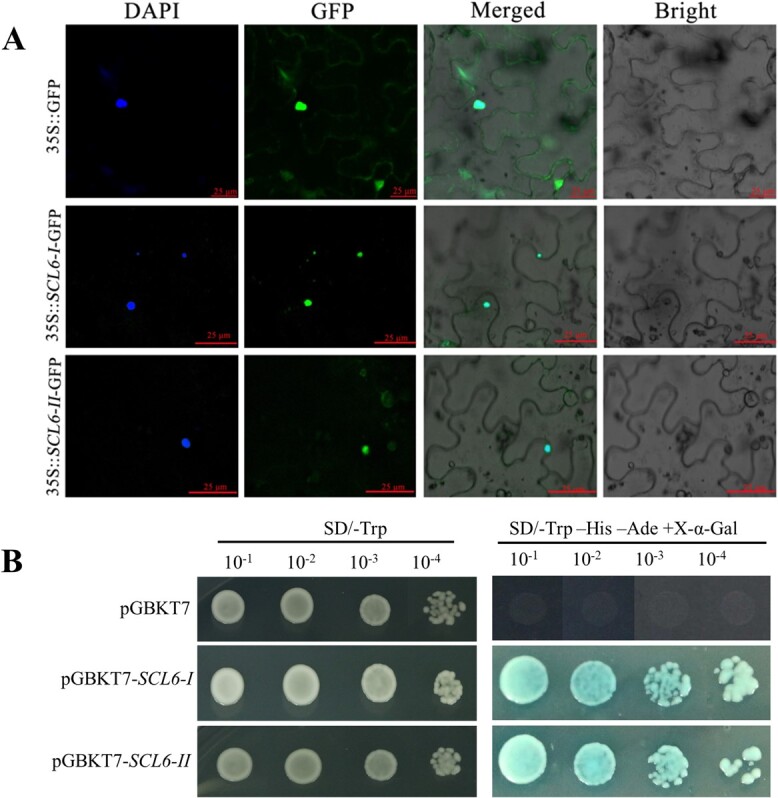
LpSCL6-I and LpSCL6-II subcellular localization and yeast transcriptional activity analysis. **a** LpSCL6-I and LpSCL6-II subcellular localization. DAPI, positive control for nuclear localization; GFP, green fluorescence channel; Merged, mixed field (superimposed photos); Bright, bright field. **b** LpSCL6-I and LpSCL6-II yeast transcriptional activity analysis. The left picture shows the result on a deficient medium (SD/−Trp) after the transformation of yeast. The right picture shows the result on triple-deficient medium (SD/−Ade/−His/−Trp) after the transformation of yeast.

pRI-SCL6-I-GFP and pRI-SCL6-II-GFP were introduced into tobacco leaves to determine the subcellular localization of LpSCL6-I and LpSCL6-II. As shown in Fig. 2a, the pRI-SCL6-I-GFP and pRI-SCL6-II-GFP fusion proteins were localized in the nucleus and overlapped with the nuclear localization signal from DAPI, but the GFP (green fluorescent protein) signal of the positive control was distributed throughout the entire cell. Colocalization of DAPI with SCL-GFP indicated the localization of SCL in the nucleus. These results suggested that both the LpSCL6-I and LpSCL6-II proteins are nuclear proteins.

The transcriptional activity test results are shown in Fig. 2b. Yeast containing the pGBKT7 empty vector grew only on SD/−Trp medium, while yeast containing pGBKT7-LpSCL6-II and pGBKT7-LpSCL6-I fusion vectors grew normally on both SD/−Trp medium and SD/−Trp−His−Ade medium. After adding X-α-Gal, the cells appeared blue, indicating α-galactosidase activity. These results indicated that LpSCL6-I and LpSCL6-II are transcriptional activators.

### Generation and expression analysis of lpu-miR171a, lpu-miR171b, *LpSCL6-II*, and *LpSCL6-I* transgenic lines

**Figure 3:**
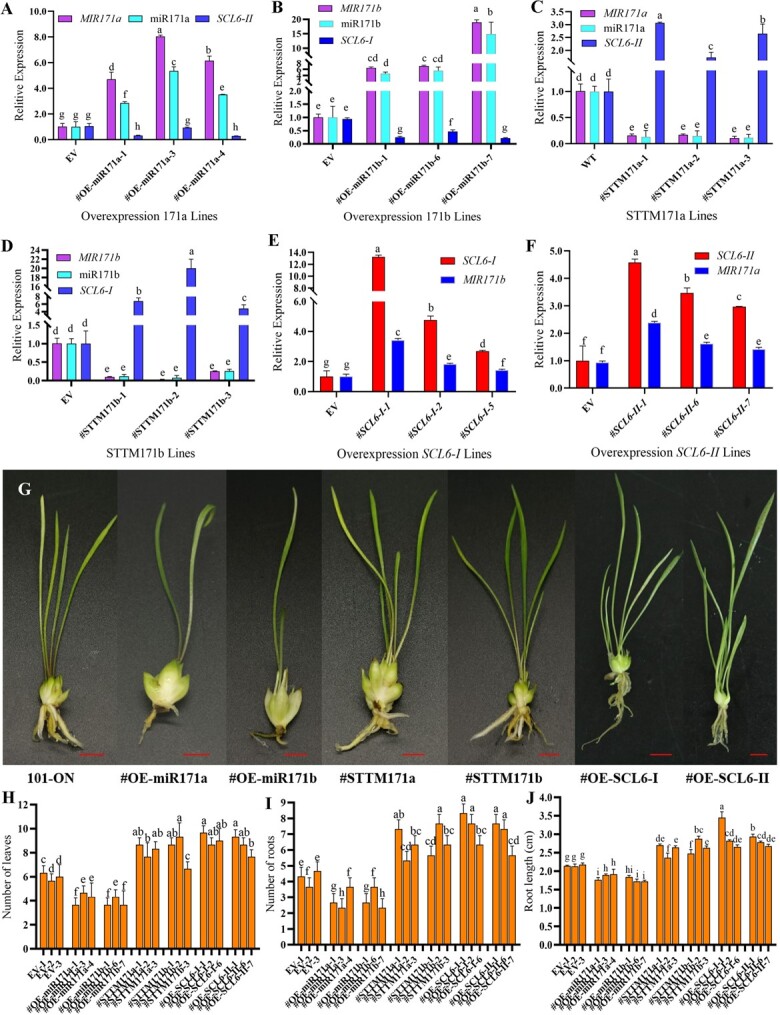
Expression detection, phenotypic observation and growth index determination in transgenic lines. **a** Expression levels of *MIR171a*, miR171a, and the target gene *LpSCL6-II* in miR171a-overexpressing lines. **b** Expression levels of *MIR171b*, miR171b, and the target gene *LpSCL6-I* in miR171b-overexpressing lines. **c** Expression levels of miR171a and the target gene *LpSCL6-II* in STTM171a-silenced transgenic lines. **d** Expression levels of miR171b and the target gene *LpSCL6-I* in STTM171b-silenced transgenic lines. **e** Expression level of *LpSCL6-I* and precursor *MIR171b* in *LpSCL6-I*-overexpressing lines. **f** Expression level of *LpSCL6-II* and precursor MIR171a in *LpSCL6-II*-overexpressing lines. **g** Phenotypic characteristics of different transgenic plants. Scale bars = 1 cm. **h** Leaf numbers of different transgenic plants. **i** Root numbers of different transgenic plants. **j** Root lengths of different transgenic plants.

qRT–PCR was used to detect target gene expression levels in plants with lpu-miR171a and lpu-miR171b overexpression or silencing to screen transgenic lines. The same method was used to screen *LpSCL6-II* and *LpSCL6-I* overexpression lines. The identification of precursors is a prerequisite for the identification of mature miRNAs. Therefore, the expression levels of lpu-miR171a and lpu-miR171b precursor and mature forms were analyzed. The results showed that the expression of miR171a and miR171b in the lpu-miR171a- and lpu-miR171b-overexpressing transgenic lines was significantly increased (Fig. 3a and b), whereas the expression of miR171a and miR171b in the silenced transgenic lines was significantly inhibited (Fig. 3c and d). The expression trends of precursor miR171a and miR171b and mature miR171a and miR171b were consistent. The lpu-miR171a- and lpu-miR171b-overexpressing and lpu-miR171a- and lpu-miR171b-silenced transgenic lines were next evaluated to determine the expression levels of *LpSCL6-II* and *LpSCL6-I*. The relative expression levels of *LpSCL6-II* and *LpSCL6-I* in the lpu-miR171a- and lpu-miR171b-overexpressing transgenic lines were significantly suppressed (Fig. 3a and b), while the relative expression levels of the target genes in the silenced transgenic lines were significantly upregulated (Fig. 3c and d).

In the *LpSCL6-II-* and *LpSCL6-I-*overexpressing transgenic lines, the expression levels of *LpSCL6-II* and *LpSCL6-I* were significantly increased (Fig. 3e and f). Subsequently, the expression levels of miR171a and miR171b in *LpSCL6-II-* and *LpSCL6-I*-overexpressing lines were evaluated. Upregulation of *LpSCL6-II* and *LpSCL6-I* caused the accumulation of lpu-miR171a and lpu-miR171b, which was significantly upregulated relative to the control (Fig. 3e and f). These results indicated that overexpression of *LpSCL6-II* and *LpSCL6-I* affects the transcription of lpu-miR171a and lpu-miR171b. In addition, these findings indicated that in *Lilium* lpu-miR171a and lpu-miR171b cleave the target genes *LpSCL6-II* and *LpSCL6-I*, thereby inhibiting their expression, and that a positive feedback regulation relationship exists between lpu-miR171a and lpu-miR171b and the target genes *LpSCL6-II* and *LpSCL6-I*.

The lpu-miR171a- and lpu-miR171b-overexpressing transgenic lines generally showed blockage of root system development and a markedly reduced number of leaves. In contrast, the lpu-miR171a- and lpu-miR171b-silenced and *LpSCL6-II-* and *LpSCL6-I*-overexpressing lines showed strong roots and many leaves (Fig. 3g). One month after the transgenic lines were transferred, the numbers of leaves and roots and root system length were quantified. Nine plants from each transgenic line were counted. The number of leaves, the root coefficient, and root system length were significantly different between the transgenic plants and the control (Fig. 3h–j).

### Lpu-miR171a and lpu-miR171b silencing or *LpSCL6-II* and *LpSCL6-I* overexpression promotes the formation of *Lilium* somatic embryos

**Figure 4:**
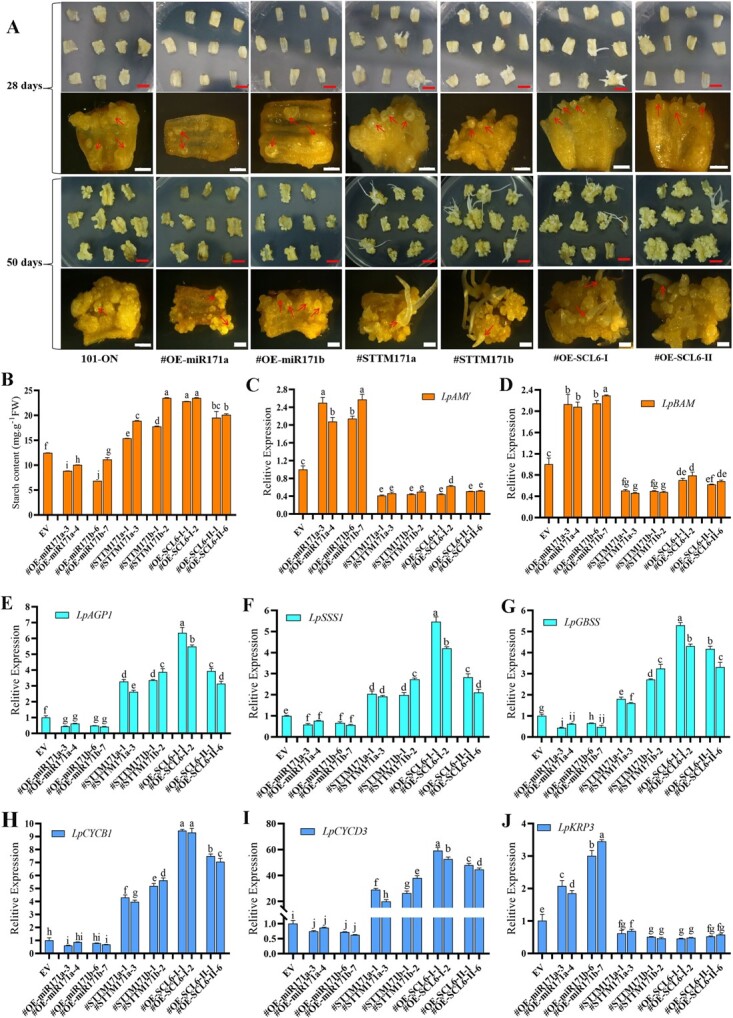
Comparison of somatic embryo induction, tissue morphology observations, starch content determination, and related gene expression. **a** Growth status of scales at 28 and 50 days of induction. The top images are photographs, and the bottom images are micrographs. Red scale bars = 0.5 cm; white scale bars = 2 mm. Red arrows point to somatic embryos. **b** Determination of starch contents in different transgenic plants. **c** and **d** Expression levels of *LpAMY* and *LpBAM*, encoding key starch metabolism enzymes. **e** Expression level of *LpAGP1*, a key starch synthesis gene. **f** Expression level of *LpSSS1*, a key starch synthesis gene. **g** Starch synthase *LpGBSS* expression. **h** and **i** Expression of the key cell cycle genes *LpCYCB1* and *LpCYCD3*. **j** Expression of the cell cycle inhibitor gene *LpKRP3*.

Somatic embryo induction was performed in different transgenic lines. At 28 days, we observed that, in contrast to the control, the lpu-miR171a- and lpu-miR1711b-overexpressing scale surfaces did not produce obvious calli. When viewed under a microscope, obvious globular embryo protrusions were observed around the vascular tissue of the scales. However, the scales of STTM171a- and STTM171b-silenced and *LpSCL6-II-* and *LpSCL6-I*-overexpressing lines formed obvious calli, and globular embryos were observed on the surface. In addition, many somatic embryos germinated and formed obvious cotyledon-shaped embryos. At 50 days, relative to the control, lpu-miR171a- and lpu-miR171b-overexpressing scales produced a small number of somatic embryos, and no somatic embryo germination leading to the formation of cotyledon-shaped embryos was observed. However, the scales of the STTM171a- and STTM171b-silenced and *LpSCL6-II-* and *LpSCL6-I*-overexpressing lines formed abundant calli and produced more somatic embryos, and more of the somatic embryos germinated and became seedlings (Fig. 4a). The results of the statistical analysis of the SE efficiency of different transgenic lines were consistent with the observation results, as shown in Supplementary Data Table S1. These results indicated that lpu-miR171a and lpu-miR171b overexpression inhibits the formation and development of somatic embryos and reduces the efficiency of SE. In contrast, these results indicated that silencing lpu-miR171a and lpu-miR171b or overexpressing *LpSCL6-II* and *LpSCL6-I* accelerates the formation of somatic embryos, increases the rate of cotyledon-type embryo formation, and significantly shortens the development time of somatic embryos.

**Figure 5:**
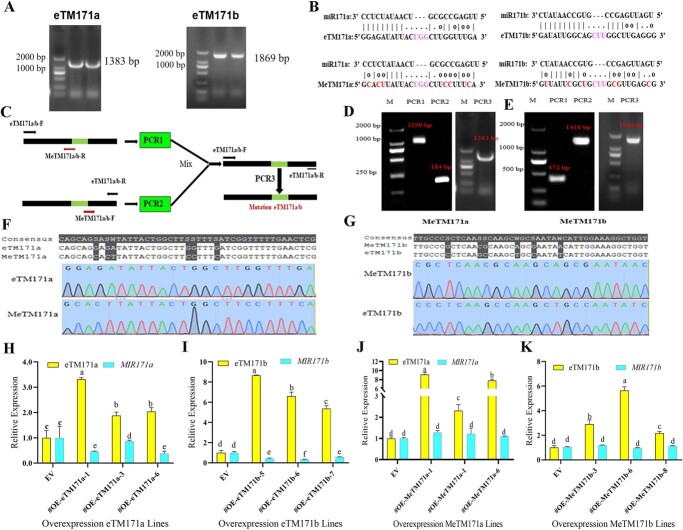
eTM171 acts as an endogenous target mimic to inhibit the expression of lpu-miR171. **a** PCR amplification product of lpu-eTM171a and lpu-eTM171b full-length cDNA (1383 and 1869 bp). **b** The mutated base sequences introduced by lpu-miR171a and lpu-miR171b. **c** The overlapping PCR method. **d** and **e** lpu-MeTM171a and lpu-MeTM171b: two rounds of PCR amplification products. **f** and **g** Sequence alignment of lpu-eTM171a and lpu-eTM171b and of lpu-MeTM171a and lpu-MeTM171b. **h** and **j** Expression level of the *MIR171a* precursor in lines with lpu-eTM171a overexpression and point mutation overexpression. **i** and **k** Expression level of the *MIR171b* precursor in lines with lpu-eTM171b overexpression and point mutation overexpression.

According to histomorphological observations, the number of embryonic cells in lpu-miR171a- and lpu-miR171b-overexpressing were lower than those in the controls at 28 days and 50 days, and few starch granules were observed. However, obvious embryonic cells were observed in the cells of the lpu-miR171a- and lpu-miR171b-silenced and *LpSCL6-II*- and *LpSCL6-I*-overexpressing lines. In addition, much larger and more abundant amyloplasts accumulated (Supplementary Data Fig. S2). Starch content measurements showed that the starch contents of the lpu-miR171a- and lpu-miR171b-overexpressing lines were lower than that of the control, and the starch content was significantly increased after lpu-miR171a and lpu-miR171b silencing and *LpSCL6-II* and *LpSCL6-I* overexpression (Fig. 4b). The expression levels of the key starch synthase genes *LpAGP1*, *LpSSS1*, and *LpGBSS* as well as the starch metabolism enzyme genes *LpAMY* and *LpBAM* were determined. The results showed that *LpAMY* and *LpBAM* were upregulated in lpu-miR171a- and lpu-miR171ab-overexpressing lines and significantly downregulated in lpu-miR171a- and lpu-miR171b-silenced and *LpSCL6-II-* and *LpSCL6-I*-overexpressing lines (Fig. 4c and d). After lpu-miR171a and lpu-miR171b overexpression, *LpAGP*, *LpSSS*, and *LpGBSS* were downregulated, whereas their expression levels were significantly upregulated after lpu-miR171a and lpu-miR171b silencing and *LpSCL6-II* and *LpSCL6-I* overexpression (Fig. 4e–g). These changes are conducive to maintaining the starch content in accordance with the results of the tissue morphological observation.

Since the somatic embryo formation cycles of the different transgenic lines were different, we evaluated the cell cycle-related gene expression of somatic embryos of different lines. The results showed that lpu-miR171a and lpu-miR171b silencing and *LpSCL6-II* and *LpSCL6-I* overexpression were accompanied by significant upregulation of the key cell cycle genes *LpCYCB1* and *LpCYCD3*. In particular, *LpCYCD3* was upregulated 40-fold in the somatic embryos of lpu-miR171b-silenced lines relative to the control, and *LpCYCD3* was upregulated 60-fold in the somatic embryos of *LpSCL6-I*-overexpressing lines relative to the control. However, the levels of *LpCYCB1* and *LpCYCD3* in the lpu-miR171a- and lpu-miR171b-overexpressing lines were slightly decreased relative to the control (Fig. 4h and i). The cell cycle suppressor gene *LpKRP3* was significantly upregulated in the lpu-miR171a- and lpu-miR171b-overexpressing lines and significantly downregulated in the lpu-miR171a- and lpu-miR171b-silenced and *LpSCL6-II-* and *LpSCL6-I*-overexpressing lines (Fig. 4j). These results further confirmed that lpu-miR171a and lpu-miR171b silencing and *LpSCL6-II* and *LpSCL6-I* overexpression accelerate the formation of somatic embryos and significantly shorten the developmental cycle of somatic embryos.

### eTM171 acts as an miRNA decoy to suppress the expression of lpu-miR171

According to the full-length cDNA sequence information of the candidate unigene40819 and unigene40253, specific fragments of 1383 bp (unigene40819) and 1869 bp (unigene40253) were amplified (Fig. 5a). To indicate their relationships with miR171a and miR171b, the cloned unigene40819 and unigene40253 were renamed lpu-eTM171a and lpu-eTM171b, respectively. Overlapping PCR was used to introduce synonymous point mutations at six bases in the lpu-eTM171a and lpu-eTM171b sequences paired with lpu-miR171a and lpu-miR171b (Fig. 5b). After three rounds of PCR (Fig. 5c), the sequences of lpu-MeTM171a and lpu-MeTM171b were amplified (Fig. 5d and e). The sequencing results showed that six bases were mutated in the lpu-eTM171a and lpu-eTM171b sequences (Fig. 5f and g). The lpu-eTM171a, lpu-eTM171b, lpu-MeTM171a, and lpu-MeTM171b overexpression vectors were constructed and transformed into *Lilium*, and overexpression lines with significantly up-regulated expression were selected by qRT–PCR (Fig. 5h–k).

To verify whether lpu-eTM171a and lpu-eTM171b affect *Lilium* SE, somatic embryo induction was performed on different transgenic lines. The results showed that at 28 days the somatic embryo induction rate and somatic embryo growth status of the lpu-eTM171a- and lpu-eTM171b-overexpressing lines and the lpu-MeTM171a and lpu-MeTM171b point mutation-overexpressing lines were not significantly different from those of the control. At 50 days, only a small number of somatic embryos germinated and formed cotyledon-shaped embryos among the somatic embryos of the lpu-eTM171b-overexpressing lines (Supplementary Data Table S1).

The expression levels of lpu-miR171a and lpu-miR171b in lpu-eTM171a and lpu-eTM171b and point mutation-overexpressing lines were detected. The results showed that the expression of miR171a and miR171b in the lpu-eTM171a- and lpu-eTM171b-overexpressing lines was significantly downregulated by ~50% (Fig. 5h and i). However, the point mutation-overexpressing (OE-MeTM171a and OE-MeTM171b) lines did not show changes in the expression of miR171a and miR171b (Fig. 5j and k). The above results demonstrated that high lpu-eTM171a and lpu-eTM171b expression has a negative regulatory effect on the corresponding lpu-miR171a and lpu-miR171b targets. However, high expression of the lpu-MeTM171a and lpu-MeTM171b point mutants had no negative regulatory effect on lpu-miR171a and lpu-miR171b, indicating that the sequence structure that binds to miRNA inhibits miRNA accumulation.

### Mode of eTM171-miR171-*SCL6* involvement in *Lilium* somatic embryogenesis

**Figure 6:**
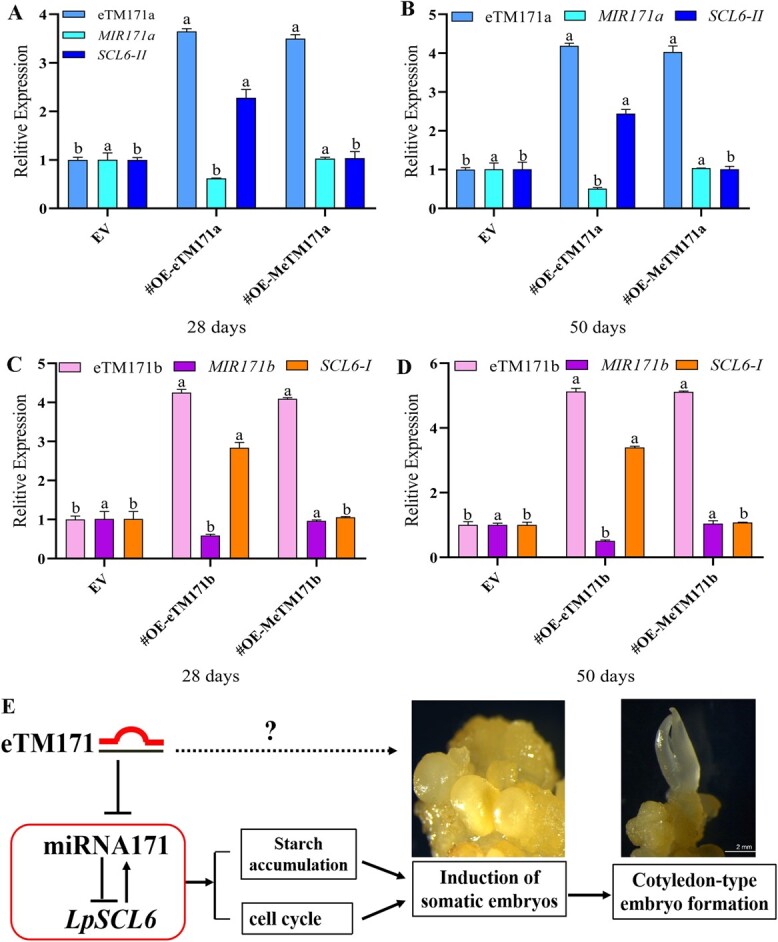
Hypothetical model of the eTM-miR171-*SCL6* module regulating SE in *L. pumilum*. **a** and **b** Expression levels of MIR171a and *LpSCL6-II* on the 28th and 50th days of lpu-eTM171a overexpression and point mutant line somatic embryo induction. **c** and **d** Expression levels of MIR171b and *LpSCL6-I* on the 28th and 50th days of lpu-eTM171b overexpression and point mutant line somatic embryo induction. Different lower-case letters in a-d indicate significant differences (tested by ANOVA) with respect to the same gene in different transgenic lines as a group. **e** Hypothetical model of the eTM-miR171-*SCL6* module regulating SE in *L. pumilum*. The solid and dotted arrows indicate positive regulation, and the inverted T-shape indicates negative regulation. The solid lines are the results obtained from the verification of this research, and the dotted lines are the results of speculation based on model plant research but not verified.

We examined the expression of lpu-miR171 and *LpSCL6* during SE in lpu-eTM171a and lpu-eTM171b and point mutation-overexpressing lines. The results showed that after 28 and 50 days of somatic embryo induction the expression of lpu-miR171a and lpu-miR171b in the OE-eTM171a and OE-eTM171b lines was downregulated, and the expression of the *LpSCL6-II* and *LpSCL6-I* target genes was significantly increased. The expression of lpu-miR171a and lpu-miR171b and the *LpSCL6-II* and *LpSCL6-I* target genes in the OE-MeTM171a and OE-MeTM171b lines was not changed significantly relative to the control (Fig. 6a–d). These results demonstrated that lpu-eTM171 acts as a decoy for lpu-miR171 and binds lpu-miR171 to prevent cleavage of the *LpSCL6* target gene.

## Discussion

Because miRNA has no coding function, it only functions by cleaving target genes or inhibiting target gene translation. In plants, RLM 5′ RACE technology is mainly used to verify the interactions between miRNAs and target genes [33], but this method cannot intuitively reflect the interaction between miRNAs and target genes. *Agrobacterium*-mediated tobacco transformation results in high transient expression efficiency and a long expression time, making this approach suitable for the study of gene interactions in plants [34]. This method is mainly used to verify the interaction between miRNAs and target genes by detecting the expression of the GUS gene in tobacco after transiently expressing miRNAs and target genes [35, 36]. In the present study, before verifying the function of lpu-miR171a and lpu-miR171b and their target genes, we used the tobacco transient transformation method to verify the interaction between lpu-miR171a and lpu-miR171b and the *LpSCL6-II* and *LpSCL6-I* target genes. GUS histochemical staining and enzyme activity test results showed that the corresponding miRNA inhibited its target gene, but the inhibition efficiency was not 100%. There may be two reasons for this result. First, there is a balanced relationship between miRNA cleavage and target gene transcription, and this relationship is strongly affected by miRNA expression levels [37]. Second, only part of the target mRNA is cleaved and degraded by miRNA, while the remaining target mRNA is separated from the cleavage system and transcribed normally [38].

The miR171-*SCL6* module is involved in branch formation [39], meristem maintenance [15], root growth, leaf formation [40], and SE [8]. The miR171 miRNA family is highly conserved. In *Arabidopsis*, three *MIR171* genes (a, b, and c) are predicted to regulate three *SCL6* genes (*SCLII*, *SCLIII*, and *SCLIV*) [41–43]. The expression domains of miR171 family members overlap with the mRNA sequences of *SCL6-II* and *SCL6-III*, indicating that both miRNAs and target mRNAs have redundant functions [44]. In *Arabidopsis*, plants overexpressing miR171c (OE171c) and triple *scl6* mutants show similar pleiotropic phenotypes, including reduced branch numbers, plant height, chlorophyll accumulation, shortened main roots, and changes in flower structure and leaf shape. These results indicate that miR171 mainly suppresses the expression of the *SCL6* gene to control extensive developmental processes during bud development [39]. However, the functions of *Lilium* miR171s are unclear.

miR171s play diverse roles in different plant species [14]. In rice, osa-miR171c accumulated substantially under abiotic stress, and transgenic lines overexpressing osa-miR171c displayed increased sensitivity to salt stress [45]. Both knockout of mdm-miR171i and overexpression of *MsSCL26.1* improved drought stress tolerance in the cultivated apple line ‘GL-3’ [46]. In this study, after lpu-miR171a and lpu-miR171b overexpression in *L. pumilum*, the plants generally showed hindered root development and fewer leaves. In contrast, the silenced plants showed strong roots and many leaves, similar to the phenotype observed in *Arabidopsis* [39] and tomato plants [20]. The expression analysis of the *LpSCL6-II* and *LpSCL6-I* target genes in transgenic lines with lpu-miR171a and lpu-miR171b overexpression and STTM silencing showed that, after lpu-miR171a and lpu-miR171b overexpression, the expression of the target genes was significantly downregulated. However, the silencing of lpu-miR171a and lpu-miR171b relieved the inhibitory effect on the target genes and significantly increased the expression of *LpSCL6-II* and *LpSCL6-I*. These findings further demonstrated that miR171a and miR171b regulate the growth and development of *Lilium* through the targeted regulation of the *SCL6* target gene.

Our previous studies have shown that miR171a and miR171b accumulate in large amounts in the early and late stages of somatic embryogenesis [13]. In this study, we found that lpu-miR171a and lpu-miR171b overexpression inhibited the formation and development of somatic embryos, and the efficiency of SE was low. In contrast, lpu-miR171a and lpu-miR171b silencing accelerated the formation of somatic embryos and significantly shortened the development of somatic embryos. The above research results indicate that in lily plants the accumulation of miR171 has a negative regulatory effect on plant growth and somatic embryo formation. miR171 was induced by abiotic stress in embryogenic callus tissues from *Larix leptolepis* [47]. In this study, plant growth and somatic embryo development were blocked after miR171 overexpression, which may be related to abiotic stress, but the relationship between miR171 and somatic embryo development under abiotic stress needs further study. The functions of miR171a and miR171b are determined by the functions of the corresponding target genes *SCL6-II* and *SCL6-I*. We found that there were 12 *SCL6* members in the lily SE transcriptome database, but only *LpSCL6-II* and *LpSCL6-I* were the target genes corresponding to miR171a and miR171b [13]. In this study, some of the functions of *LpSCL6-II* and *LpSCL6-I* were similar, while others were different. Both genes play important roles in the early and late stages of somatic embryogenesis; however, *LpSCL6-I* has a more obvious effect than *LpSCL6-II*.

*CYCB* and *CYCD* are key genes in the G2/M transition that are often used as markers of cell division [48]. Cell cycle repressor kip-related proteins (KRPs) mainly inhibit cell cycle proteins and negatively regulate cell proliferation [49]. In this study, the expression levels of *LpCYCB1* and *LpCYCD3* were significantly upregulated after *LpSCL6* overexpression, while the expression of the *LpKRP* gene was inhibited, indicating that *LpSCL6* overexpression promoted the proliferation of *Lilium* embryogenic cells and was beneficial to the formation of somatic embryos. Studies have shown that the increase in the contents of embryogenic calli, especially amyloplasts, can provide an energy and material basis for the formation of somatic embryos [5]. In this study, histomorphological observations showed that few amyloplasts accumulated in embryonic cells overexpressing lpu-miR171a and lpu-miR171b compared with the control lines. However, lpu-miR171a and lpu-miR171b silencing and *LpSCL6-II* and *LpSCL6-I* overexpression resulted in the accumulation of much larger and more abundant amyloplasts. In *Carica papaya*, increased sucrose and starch content is a key point in somatic embryo transformation, indicating that carbohydrates such as starch play a key role in the morphogenesis of somatic embryos [50]. Among the key enzymes in starch synthesis, adenosine 5′-diphosphate glucose pyrophosphorylase (AGPase) is considered to be the key enzyme required for the first step of plant starch synthesis [51]. Starch synthase (SS) includes granule-bound starch synthase (GBSS) and soluble starch synthase (SSS). GBSS mainly catalyzes the synthesis of amylose, and SSS mainly catalyzes the synthesis of amylopectin [52]. α-Amylase (AMY) and β-amylase (BAM) are key enzymes in starch degradation [53]. In this study, overexpression of *LpSCL6* promoted the expression of starch synthase genes *LpAGP*, *LpSSS* and *LpGBSS*, and inhibited the expression of starch degradation genes *LpAMY* and *LpBAM*. These results were consistent with the previous conclusion that overexpression of *LpSCL6* promoted the accumulation of starch.

Tang *et al*. [28] developed STTM technology based on TMs, which is an effective tool for studying miRNA functions in plants. The overexpression of STTMs has been used to identify miRNA functions in a variety of plants, such as *Arabidopsis* and wheat [54, 55]. To further explore the function of lpu-miR171a and lpu-miR171b candidates, we used STTM technology to silence endogenous miRNAs based on stable transformation. The results showed that lpu-miR171a and lpu-miR171b silencing effectively shortened the SE cycle of *L. pumilum*, thereby revealing a new function of miR171 in the regulation of *Lilium* and plant growth and development.

The present study confirmed the function of lpu-miR171 and the *LpSCL6* target gene in the SE of *L. pumilum* and elucidated the regulatory effect of lpu-miR171 on the *LpSCL6* target gene. In summary, a hypothetical model whereby the eTM-miR171-*SCL6* module regulates SE in *L. pumilum* is proposed based on combining our results and previous reports involving model plants (Fig. 6e). Our research provides promising candidate genes for enhancing the SE ability of *Lilium* and lays a good foundation for studying the mechanism of SE in this genus.

## Materials and methods

### Plant materials

Embryogenic calli and somatic embryos of *L. pumilum* DC. Fisch. were obtained according to a previously described method [27]. Tobacco seedlings aged 28 days were also used.

### Gene cloning

Genomic DNA was extracted via the CTAB method as described previously [26]. According to the predicted sequence information on the lpu-eTM171 candidate, Primer 5.0 software was used to design primers (shown in Supplementary Data Table S2), and the DNA was used as a template for PCR amplification. The PCR products were inserted into the pMD18-T vector and sequenced. Sequence alignment was performed using DNAMAN8.

### Plasmid construction and *Lilium* transformation

The precursor fragments of lpu-miR171a and lpu-miR171b and the coding sequence (CDS) regions of *LpSCL6-II* and *LpSCL6-I* and lpu-eTM171a and lpu-eTM171b were amplified by PCR. According to the seamless cloning principle of the In-Fusion HD Cloning Kit (TaKaRa, Dalian, China), these products were cloned into the plasmid pRI101-ON. A short tandem target mimic (STTM) sequence (Supplementary Data Table S2) that specifically inhibited lpu-miR171a and lpu-miR171b was designed and synthesized according to the method of Tang *et al*. [28] and cloned into the pRI101-ON plasmid. A schematic diagram of the vector structure is shown in Supplementary Data Fig. S1. All of the above vectors were used to transform the embryogenic calli of *L. pumilum*. For genetic transformation, the method that we reported previously was used [29]. The precursor fragments of lpu-miR171a and lpu-miR171b and the CDS regions of *LpSCL6-II* and *LpSCL6-I* were cloned into the pRI101-ON-GUS and pRI-ON-LUC plasmids for the transient transformation of tobacco leaves.

We introduced a six-base synonymous point mutation into the lpu-eTM171a and lpu-eTM171b sequences paired with lpu-miR171a and lpu-miR171b by referring to the reported method of lncRNA23468 point mutation in tomato [30]. The lpu-eTM171a and lpu-eTM171b mutations were generated by overlapping PCR, which involved the use of lpu-eTM171a and lpu-eTM171b as templates for amplification and mutagenesis. Three rounds of PCR were performed to amplify the lpu-MeTM171a and lpu-MeTM171b mutants (the procedure is illustrated in Fig. 5c). Point mutation overexpression vectors were constructed (named pRI101-MeTM171a and pRI101-MeTM171b) and transformed into embryogenic calli of *Lilium* (the primers used are shown in Supplementary Data Table S2).

### β-Glucuronidase histochemical staining and luciferase reporter system detection

β-Glucuronidase (GUS) histochemical staining and enzyme activity detection were performed as described previously [26]. The pRI-mini35S-LUC, 35S::LUC-SCL6 I, 35S::LUC-SCL6 II, pRI-101-miR171a, and pRI-101-miR171b luciferase (LUC) reporter gene vectors as well as the coexpression vectors (35S::miR171a + 35S::LUC-SCL6 II and 35S::miR171b + 35S::LUC-SCL6 I) were transferred into tobacco leaves and observed with a digital *in vivo* luminescence imager after 48 h.

### Subcellular localization and transcriptional activation analysis

The full-length *LpSCL6-II* and *LpSCL6-I* CDSs without the termination codon were amplified in the pRI101-GFP vector. The primers used for this purpose are shown in Supplementary Data Table S2. Using the DAPI (4′,6-diamidino-2-phenylindole) nuclear localization dye signal as a positive control and pRI-101-GFP as a negative control, the vectors were injected into *Nicotiana benthamiana* leaves. After transformation for 2 days, the leaves were visualized using a laser confocal fluorescence microscope (TCSSP8-SE, Leica, Wetzlar, Germany).

The transactivation assay was performed using the Matchmaker GAL4 Two-Hybrid System 3 (Clontech, Palo Alto, CA, USA). The coding regions of *LpSCL6-II* and *LpSCL6-I* were inserted into the pGBKT7 vector. The pGBKT7-LpSCL6-I and pGBKT7-LpSCL6-II constructs as well as the pGBKT7 negative control were transformed into the Y2H Gold yeast strain. The transformed strains were grown on SD/−Trp medium and selected on SD/−Trp−His−Ade medium.

### Gene expression analysis

Leaves and calli of the control and transgenic lilies were collected separately and stored at −80°C. The HiPure HP Plant RNA Kit (Magen, Shanghai, China) was used to extract total RNA, and the FastKing cDNA Kit (Tiangen, Beijing, China) was used to reverse-transcribe the RNA into cDNA. Referring to our previous report [31], a QuantStudio 3 Real-Time PCR System was used for qRT–PCR analysis. The qRT–PCR primers are listed in Supplementary Data Table S2. Gene expression levels were calculated using the 2^–ΔΔCt^ method. Each sample included three biological replicates and three technical replicates.

### Comparison of somatic embryo inducibility

In the SE system, somatic embryo induction medium was inoculated with different transgenic lines of *Lilium*. Transgenic lines carrying empty vectors were used as controls to compare the SE and regeneration efficiency of the transgenic lines. The process of somatic embryogenesis was observed, and the somatic embryogenesis induction rate and cotyledon-shaped embryo induction rate were calculated at 28 and 50 days. Each experiment included 30 explants and was carried out three times. The SE induction rate (%) was calculated as $\frac{\ \mathrm{No}.\mathrm{of}\ \mathrm{explants}\ \mathrm{forming}\ \mathrm{somatic}\ \mathrm{embryos}\ }{\mathrm{No}.\mathrm{of}\ \mathrm{explants}\ \mathrm{used}}\times 100$. The cotyledon-shaped embryo induction rate (%) was calculated as $\frac{\ \mathrm{No}.\mathrm{of}\ \mathrm{explants}\ \mathrm{forming}\ \mathrm{cotyledon}-\mathrm{shaped}\ \mathrm{embryos}\ }{\mathrm{No}.\mathrm{of}\ \mathrm{explants}\ \mathrm{used}}\times 100$.

### Histomorphology and determination of starch content

Morphological observations of the somatic embryos induced from different transgenic lines were performed at different developmental stages. The samples were fixed in formalin–acetic acid–alcohol (FAA; 90% ethanol, 5% formaldehyde, and 5% acetic acid; v/v/v) for 24 h at 4°C, and paraffin sections were prepared according to the method of Fu *et al*. [32]. After dehydration-transparency embedding, the embedded wax blocks were sliced into 6-μm sections using a microtome (RM 2245, Leica, Germany). After staining with 0.01% toluidine blue, the sections were observed and photographed under an optical microscope (DM3000, Leica, Germany). The morphological structures of somatic embryos at different stages, somatic embryo contents, and starch granule contents were observed and compared. The starch content was determined according to the instructions of the starch determination kit (Suzhou Keming Biotechnology Co., Ltd., Suzhou, China).

### Statistical analysis

The data were analyzed with IBM SPSS Statistics 23.0 (SPSS Inc., Chicago, USA) for one-way ANOVA and Duncan’s test. The effects were considered significant at *P* < .05.

## Acknowledgements

This work was supported by the National Key R&D Program of China (2018YFD1000407), the National Natural Science Foundation of China (Grant Nos. 31672179 and 31872150) and the LiaoNing Revitalization Talents Program (XLYC2002052).

## Author contributions

R.Y. and H.M.S. conceived and designed the experiments. R.Y. and S.L.S. performed the experiments and analyzed the data. S.L.S. and H.Y.L. provided the experimental methods and participated in the discussion. R.Y. and H.M.S. wrote and revised the manuscript. R.Y. and S.L.S. contributed equally to this work. All authors read and approved the final manuscript.

## Data availability

The data that support the findings of this study are available from the corresponding author upon reasonable request.

## Conflict of interest

The authors declare that they have no conflicts of interest.

## Supplementary data

Supplementary data is available at *Horticulture Research* online.

## Supplementary Material

Web_Material_uhac045
