# Supplementary material for: Functional analysis of the eTM-miR171-SCL6 module regulating somatic embryogenesis in Lilium pumilum DC. Fisch
Source: Hortic Res. 2022 Feb 19;9:uhac045. doi: 10.1093/hr/uhac045 (PMC9171120; doi:10.1093/hr/uhac045)
Supplement: Web_Material_uhac045 [file web_material_uhac045.docx]

# Supplementary Figures


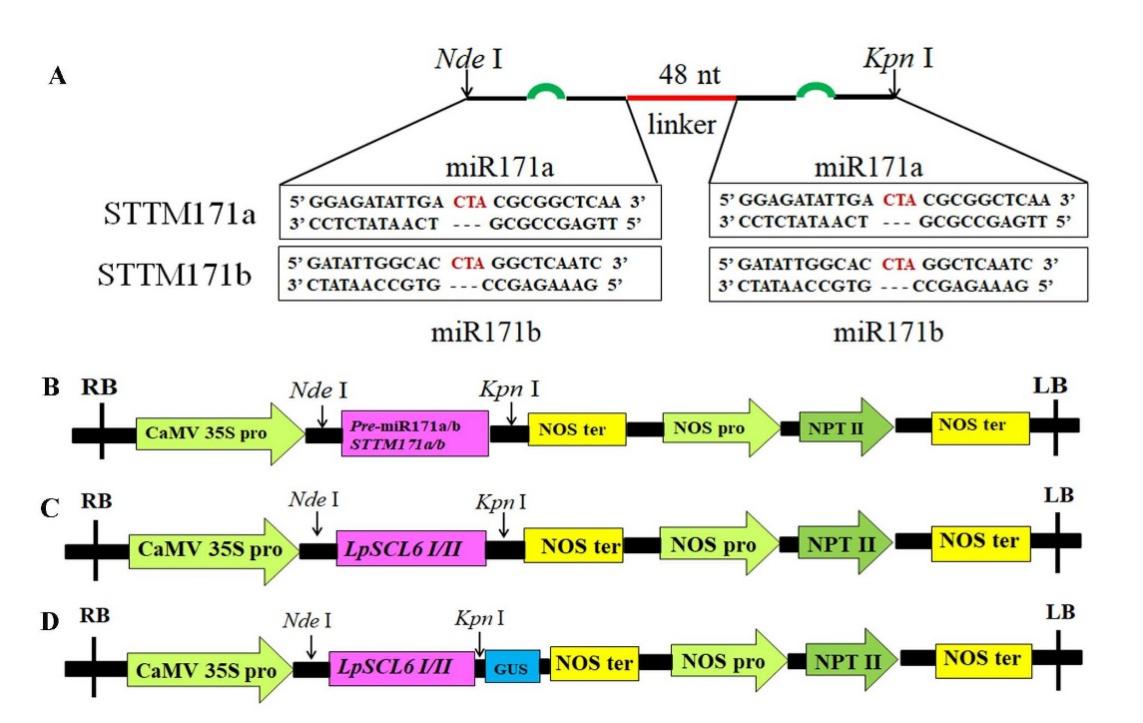


**Supplementary Fig. 1** Structure of expression vector. **A** Structure of STTM171a/b. B. Structure of lpu-miR171a/b and STTM171a/b overexpression vector. **C** pRI-SCL6-I/II vector structure (for stable transformation). **D** pRI-SCL6-I/II-GUS vector structure (for transient expression verification interaction).


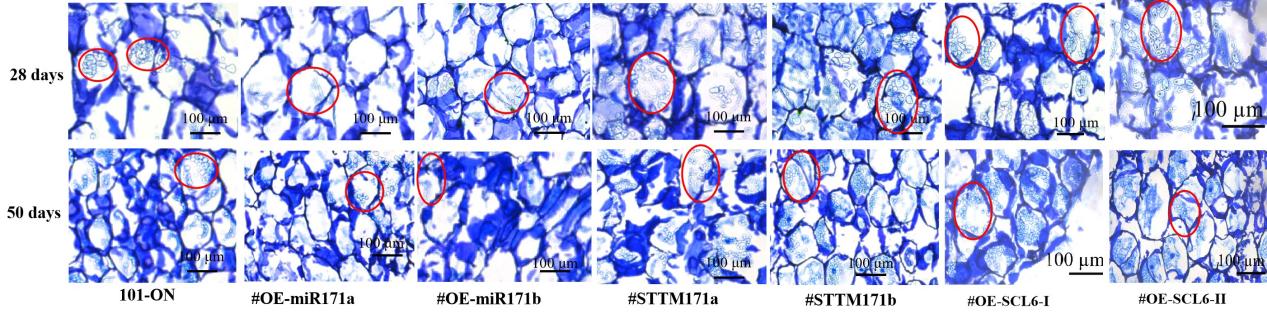


**Supplementary Fig. 2** Tissue morphology observations after 28 and 50 days of induction.

# Supplementary Tables

Supplementary Table 1 Effect of miR171a/b overexpression and STTM171a/b silences transgenic lines on the efficiency of somatic embryogenesis

| Lines | 28 days | | 50 days | |
| --- | --- | --- | --- | --- |
|  | Induction rate (%) | Cotyledon-shaped embryo rate (%) | Induction rate (%) | Cotyledon-shaped embryo rate (%) |
| #101-ON | 96.67 ± 5.77 a | 0 f | 100 a | 6.67 ± 5.77 e |
| #OE-miR171a-3 | 63.33 ± 5.77 c | 0 f | 83.33 ± 5.77 b | 3.33 ± 5.77 e |
| #OE-miR171a-4 | 66.67 ± 5.77 bc | 0 f | 80.00 ± 10.00 bc | 3.33 ± 5.77 e |
| #OE-miR171b-6 | 76.67 ± 5.77 b | 0 f | 73.33 ± 5.77 c | 3.33 ± 5.77 e |
| #OE-miR171b-7 | 76.67 ± 5.77 b | 0 f | 76.67 ± 5.77 bc | 6.67 ± 5.77 e |
| #STTM171a-1 | 90.00 ± 10.00 a | 23.33 ± 5.77 bc | 100 a | 53.33 ± 5.77 b |
| #STTM171a-3 | 93.33 ± 5.77 a | 36.67 ± 5.77 a | 96.67 ± 5.77 a | 56.67 ± 5.77 ab |
| #STTM171b-1 | 93.33 ± 5.77 a | 30 ± 10.00 ab | 96.67 ± 5.77 a | 63.33 ± 5.77 a |
| #STTM171b-2 | 96.67 ± 5.77 a | 36.67 ± 11.55 a | 100 a | 60 ab |
| #OE-*SCL6-I*-1 | 93.33 ± 5.77 a | 23.33 ± 5.77 bc | 96.67 ± 5.77 a | 40.00 ± 10.00 c |
| #OE-*SCL6-I*-2 | 96.67 ± 5.77 a | 20 c | 100 a | 43.33 ± 5.77 c |
| #OE-*SCL6-II*-1 | 93.33 ± 5.77 a | 16.67 ± 5.77 cd | 93.33 ± 5.77 a | 36.67 ± 5.77 c |
| #OE-*SCL6-II*-6 | 93.33 ± 5.77 a | 16.67 ± 5.77 cd | 96.67 ± 5.77 a | 40.00 ± 10.00 c |
| #OE-eTM171a-1 | 96.67 ± 5.77 a | 0 f | 96.67 ± 5.77 a | 0 e |
| #OE-eTM171a-6 | 100 a | 0 f | 100 a | 0 e |
| #OE-MeTM171a-1 | 96.67 ± 5.77 a | 0 f | 96.67 ± 5.77 a | 0 e |
| #OE-MeTM171a-6 | 93.33 ± 5.77 a | 0 f | 93.33 ± 5.77 a | 0 e |
| #OE-eTM171b-5 | 93.33 ± 5.77 a | 10 de | 96.67 ± 5.77 a | 16.67 ± 5.77 d |
| #OE-eTM171b-6 | 96.67 ± 5.77 a | 6.67 ± 5.77 ef | 96.67 ± 5.77 a | 13.33 ± 5.77 d |
| #OE-MeTM171b-3 | 96.67 ± 5.77 a | 0 f | 100 a | 6.67 ± 5.77 e |
| #OE-MeTM171b-6 | 96.67 ± 5.77 a | 0 f | 96.67 ± 5.77 a | 3.33 ± 5.77 e |

Data in the table represent mean ± standard error. Statistically, one way analysis of variance (ANOVA) and Duncan’s post-hoc tests were used for statistical comparisons of the somatic embryo induction rate of diferent transgenic plants. Diferent letters indicate signifcant diference within a group as determined by Student’s t test (*p*<0.05).

Supplementary Table 2 Primers used in this study

| Assay | Primers name | Sequence（5’-3’） |
| --- | --- | --- |
| Gene cloning | eTM171a-F | ATGTCCAACGAGCACGAGTACC |
|  | eTM171a-R | TCATGAGCAAGAATTCTCACAGGC |
|  | eTM171b-F | ATGGGTGAGTTCGGAAAGCG |
|  | eTM171b-R | ATGGGTGAGTTCGGAAAGCG |
|  | MeTM171a-F | TGGCTTCCTTTCATCGGTT |
|  | MeTM171a-R | CCAGTAATAAGTGCTGCTGC |
|  | MeTM171b-F | TTCTTGCCCCCTCAAGCCAA |
|  | MeTM171b-R | TTCCAATGATATTGGCAGC |
|  | SCL6-I-F | ATGCCCTTCAATCTTCAACTG |
|  | SCL6-I-R | TCAGCACCTCCACGCTGAC |
|  | SCL6-II-F | ATGCCCTTTGATTTCCAACAC |
|  | SCL6-II-R | CTGCACCTTCTTCAACAAGCAC |
| Vector construction of genetic transformation | eTM171a-O-F | GAGGGGGATTACATATGATGTCCAACGAGCACGAGTACC |
|  | eTM171a-O-R | TTGATTCAGGATCCGGTACCTCATGAGCAAGAATTCTCACAGGC |
|  | eTM171b-O-F | GAGGGGGATTACATATGATGGGTGAGTTCGGAAAGCG |
|  | eTM171b-O-F | TTGATTCAGGATCCGGTACCTTAAGTTGCCATGATAAAAGCTTGG |
|  | SCL6-I-0-F | GAGGGGGATTACATATGATGCCCTTCAATCTTCAACTGTTGG |
|  | SCL6-I-0-R | TGTTGATTCAGGATCCTCAGCACCTCCACGCTGAC |
|  | SCL6-II-0-F | GAGGGGGATTACATATGATGCCCTTTGATTTCCAACACAAGG |
|  | SCL6-II-0-R | TGTTGATTCAGGATCCCTGCACCTTCTTCAACAAGCAC |
|  | 171a-O-F | GAGGGGGATTACATATGTGAGAACAGGCGGGTGAATG |
|  | 171a-O-R | TGTTGATTCAGGATCCATTTGCAGTGCATGGGAGATATTG |
|  | 171b-O-F | GAGGGGGATTACATATGAGGTAATGTAATGCGGCTAGAACG |
|  | 171b-O-R | TGTTGATTCA GGATCCGGCACCACAGCTCGAACC |
| Sub-cellular localization | eTM171a- G-F | GAGGGGGATTA CATATG ATGTCCAACGAGCACGAGTACC |
|  | eTM171a- G-R | TGCTCACCATGGATCCTGAGCAAGAATTCTCACAGGC |
|  | eTM171b- G-F | GAGGGGGATTACATATGATGGGTGAGTTCGGAAAGCG |
|  | eTM171b- G-R | TGCTCACCATGGATCCAGTTGCCATGATAAAAGCTTGG |
|  | SCL6-I-G-F | GAGGGGGATTACATATGATGCCCTTCAATCTTCAACTGTTGG |
|  | SCL6-I-G-R | TGTTGATTCA GGATCC GCACCTCCACGCTGAC |
|  | SCL6-II-G-F | GAGGGGGATTACATATGATGCCCTTTGATTTCCAACACAAGG |
|  | SCL6-II-G-R | TGTTGATTCAGGATCCCTGCACCTTCTTCAACAAGCAC |
| Transcriptional activation analysis | SCL6-I-T-F | AGGAGGACCTGCATATGATGCCCTTCAATCTTCAACTGTTGG |
|  | SCL6-I-T-R | GCAGGTCGACGGATCCTCAGCACCTCCACGCTGAC |
|  | SCL6-II-T-F | AGGAGGACCTGCATATGATGCCCTTTGATTTCCAACACAAGG |
|  | SCL6-II-T-R | GCAGGTCGACGGATCCCTGCACCTTCTTCAACAAGCAC |
| Verification of transgenic lines | 35S | GACGCACAATCCCACTATCC |
|  | RV | CAGGAAACAGCTATGAC |
| Gene expression | FP-F | TCGCCTACATCGCTAACC |
|  | FP-R | TTCCCAATAATCGCAAGACC |
|  | eTM171a-q-F | CCAACGAGCACGAGTACCAACC |
|  | eTM171a-q-R | AGGCGAGGATTTCGGAGACA |
|  | eTM171b-q-F | GAGGTAGATAATGTGCGTGA |
|  | eTM171b-q-R | CTGCCAGAAAGCATCCC |
|  | 171a-q-F | ACACTCCAGCTGGGTTGAGCCGCGTCAAT |
|  | 171a-q-R | TGGTGTCGTGGAGTCG |
|  | 171b-q-F | ACACTCCAGCTGGGTGATTGAGCCGTGC |
|  | 171b-q-R | TGGTGTCGTGGAGTCG |
|  | SCL6-I-q-F | TCGCCGATGCAGGATTTGG |
|  | SCL6-I-q-R | GCTGAGACTGGTGGAGATGGTTGA |
|  | SCL6-II-q-F | GCCAAATCGCCACCAAATCC |
|  | SCL6-II-q-R | TCGCCGCCCGCCTTCAGTT |
|  | CYCB1-F | ATTGGACAGCTTTGGTTTCG |
|  | CYCB1-R | AAGCCAAGCAACAATTCCAT |
|  | CYCD3-F | CCCGTTCTCCCATGATCTTA |
|  | CYCD3-R | CAATGATTAAGCGGCCATCT |
|  | KRP3-F | CCAACTCGACCACTGCTACA |
|  | KRP3-R | ACTCGTAACGTCCAGGGAGA |
|  | AMY-F | CTCCCGACATCGATCATCTT |
|  | AMY-R | ATCTTATTGCGGTTGGCATC |
|  | BAM-F | AATGAATGATCCGGATGGAA |
|  | BAM-R | AAAGTTATTGTGCCGCATCC |
|  | AGP1-F | TTAGAATCTGGTGTTGAACTGAAGG |
|  | AGP1-R | TCGTCCTTGATGGTGGTGTTCTTAG |
|  | SSS1-F | GTGGTCCTGTCGGCTTTATGTTC |
|  | SSS1-R | TTGTGATCGGTCAGCTTCGC |
|  | GBSS-F | GGGATGAACTTGGTGTATGTCG |
|  | GBSS-R | TCTTATAGAGATGAAAGTAGCGCAC |
